# Analysis of *Clostridium beijerinckii* NCIMB 8052’s transcriptional response to ferulic acid and its application to enhance the strain tolerance

**DOI:** 10.1186/s13068-015-0252-9

**Published:** 2015-04-16

**Authors:** Siseon Lee, Jin Hyung Lee, Robert J Mitchell

**Affiliations:** School of Life Sciences, Ulsan National Institute of Science and Technology, 50 UNIST-gil, Eonyang-eup, Ulsan, 689-798 South Korea; Korea Institute for Ceramic Engineering and Technology, 101, Soho-ro, Jinju-si, Gyeongsangnam-do 660-031 South Korea

## Abstract

**Background:**

Plant-based cellulose presents the best source of renewable sugars for biofuel production. However, the lignin associated with plant cellulose presents a hurdle as hydrolysis of this component leads to the production of inhibitory compounds, such as ferulic acid.

**Results:**

The impacts of ferulic acid, a phenolic compound commonly found in lignin hydrolysates, on the growth, solvent production, and transcriptional responses of *Clostridium beijerinckii* NCIMB 8052 were determined. Addition of ferulic acid to growing cultures resulted in a decrease in the growth and solvent production by 30% and 25%, respectively, when compared to the control cultures. To better understand the toxicity of this compound, microarray analyses were performed using samples taken from these cultures at three different growth states. Several gene ontology terms and Kyoto Encyclopedia of Genes and Genomes (KEGG) pathways were identified showing significant change at each status, including ATP-binding cassette (ABC) transporters, two component system, and oxidoreductase activity. Moreover, genes related with efflux systems and heat shock proteins were also strongly up-regulated. Among these, expression of the *groESL* operon was induced by more than fourfold and was consequently selected to improve *C. beijerinckii* tolerance to ferulic acid. Real-time quantitative PCR (RT-qPCR) analysis confirmed that *C. beijerinckii* harboring the plasmid, pSAAT-ptb_Gro, had a two- to fivefold increased *groESL* operon expression during growth of these cultures. Moreover, this strain was more tolerant to ferulic acid as the growth of this recombinant strain and its bioconversion of glucose into solvents were both improved.

**Conclusions:**

Using transcriptomics, we identified numerous genes that are differentially expressed when *C. beijerinckii* cultures were exposed to ferulic acid for varying amounts of time. The operon expressing *groESL* was consistently up-regulated, suggesting that this gene cluster may contribute to strain tolerance. This was confirmed as recombinant cultures showed both an enhanced growth and solvent yield in the presence of 0.5 g/L ferulic acid.

**Electronic supplementary material:**

The online version of this article (doi:10.1186/s13068-015-0252-9) contains supplementary material, which is available to authorized users.

## Background

With the challenges with conventional resources, such as corn, lignocellulosic biomass has been considered as one of the promising sustainable sources for biofuel production, in that it is the most abundant non-food material with availability as a variety of types, such as crop residues, grass, and hard and soft woods [1]. Conversion of the biomass to useful biochemical would be initiated from the pretreatment process to release sugar components by disrupting the complicated structure, consisting of cellulose, hemicellulose, and lignin. During this process, however, several byproducts, including furan derivatives and phenolic compounds, can be generated, which limits the use of biomass for biofuel production due to their toxicity to fermentative bacterial strains [2,3]. Although several methods have been known to deal with those inhibitors with the use of enzyme, vacuum evaporation, and overliming [4-7], it is hard to eliminate completely from hydrolysates. In addition, they require considerable energy and extra materials while generating wastes and increasing the overall production costs. Therefore, it has been suggested, as an alternative, to develop more robust and tolerant stains to toxic hydrolysate-related compounds so that it would be possible to simultaneously ferment the sugars within the hydrolysates in the presence of inhibitory compounds.

In the past, adaptations, where the cultures experience the stress condition serially, or random mutagenesis, where the cells are exposed to mutagenic chemicals, were primarily used to make genetically modified organism [8,9]. Nowadays, the analyses of individual gene expression responding to stresses become possible with transcriptomic analysis, such as microarray analysis, which makes the result of the gene modification more accurately predictable. Based upon the analysis, one or several genes, only that we are interested in, can be selected to manipulate and construct new strain to achieve our purpose. Several studies were reported on transcriptomic analysis to determine the toxicity of solvent to bacterial strains [10,11]. Recently, the stress of ferulic acid, one of lignin-derived compounds, was evaluated within *Escherichia coli* [12] and *Lactobacillus brevis* [13]. Although biofuel, such as butanol, was produced using those strains [14,15], they were engineered to have external metabolic pathway and their substrate was limited to glucose, suggesting that non-natural biofuel-producing strains still remains under doubt about their application. However, for clostridia, natural butanol-producing strains, the analysis about the impact of the lignocellulosic biomass-derived inhibitors is still not sufficiently available. Although transcriptional analysis of *Clostridium beijerinckii* exposed to furfural has been recently reported [16], the detrimental effect of aromatic compounds arouse from lignin portion is severer than other compounds from sugar, such as furfural, at lower concentration level [2]. Moreover, it has been shown that furfural and HMF were naturally converted to less toxic alcohol type whereas the phenolic compounds including ferulic acid and syringaldehyde remained within the media [17]. Due to these reasons, more studies on the effects of phenolic acid on butanol-producing *Clostridium* strains has been demanded to develop the new strain tolerant to lignocellulosic biomass hydrolysate.

In this study, therefore, microarray analysis was conducted when *C. beijerinckii* NCIMB 8052, one of natural butanol-producing *Clostridium* strains, was exposed to ferulic acid in order to determine the effects of compound on its transcription level. To better understand the toxic mechanisms for industrial application purpose, the responses of the strain were assessed as the instant, short-, and long-term responses, according to the incubation time. Ultimately, by using the analysis results, *C. beijerinckii* was newly modified to have more production of protein chaperone to increase the ferulic acid tolerance and the change in mRNA was also investigated.

## Results and discussion

### *C. beijerinckii* NCIMB 8052 growth and solvent production under ferulic acid stress

**Figure 1:**
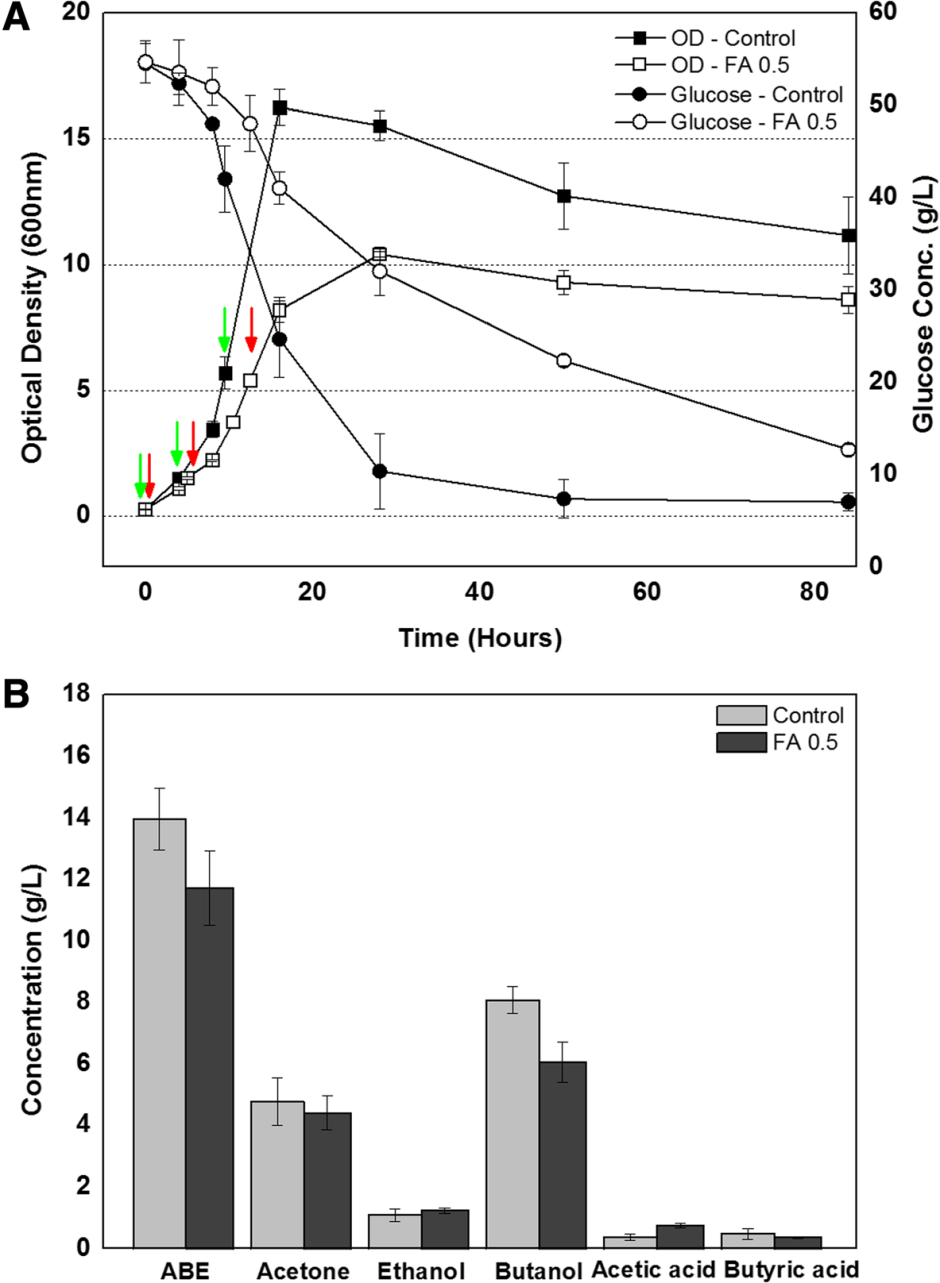
Effect of ferulic acid on *C. beijerinckii* NCIMB 8052 growth, sugar consumption **(A)** and final acid and solvent production **(B)**. Samples from both cultures were taken for RNA purification according to optical density of the culture as indicated by the arrows.

Ferulic acid was chosen as a model lignocellulosic biomass-derived compound for this study because it has detrimental effects on fermentative microbes even when present at a small concentration [2,3]. As expected, ferulic acid at a concentration of 0.5 g/L delayed the growth rate of *C. beijerinckii* NCIMB 8052 during the exponential phase by 30% (Figure 1A). In addition, the maximum optical density at 600 nm (OD600) for the culture exposed to ferulic acid was 10.4 after 28 h. By comparison, that of the control culture was 16.2 at 16 h. The slowed growth caused by ferulic acid also led that the solvent production was initiated 4 h later. The inhibitory effect of the ferulic acid on solvent production was also shown in Figure 1B. Total solvent (acetone-butanol-ethanol) and butanol concentration in the presence of ferulic acid reduced to 11.71 and 6.07 g/L, compared to 13.95 and 8.08 g/L in the absence of ferulic acid, respectively.

To better understand the mechanisms by which ferulic acid affects *C. beijerinckii* NCIMB 8052’s physiology and the resulting transcriptional response, whole genome microarray analyses were performed using the total cellular RNA prepared from cultures grown with or without 0.5 g/L of ferulic acid. Moreover, RNA was purified from three different time points according to optical density (indicated as arrows in Figure 1A) in order to investigate and compare the instant, short-, and long-term responses of this bacterium to ferulic acid.

### RT-qPCR validation of microarray gene expression results

**Figure 2:**
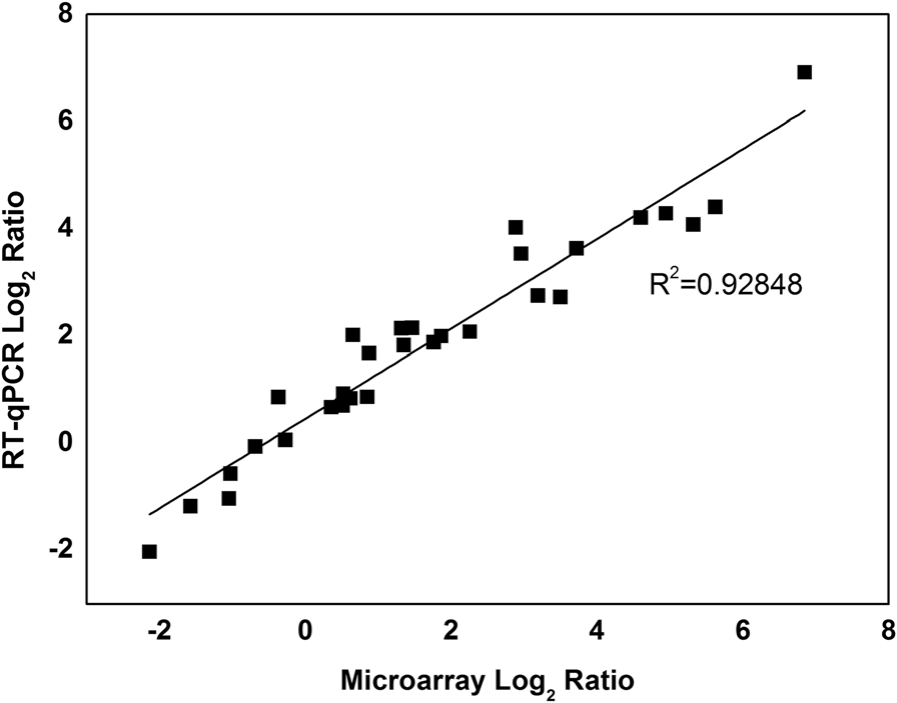
Validation of the microarray values by RT-qPCR. This figure shows the relative expression levels from the microarray analysis plotted against those obtained by RT-qPCR. The good *R* ^2^ value indicates that the microarray values are accurate.

**Figure 3:**
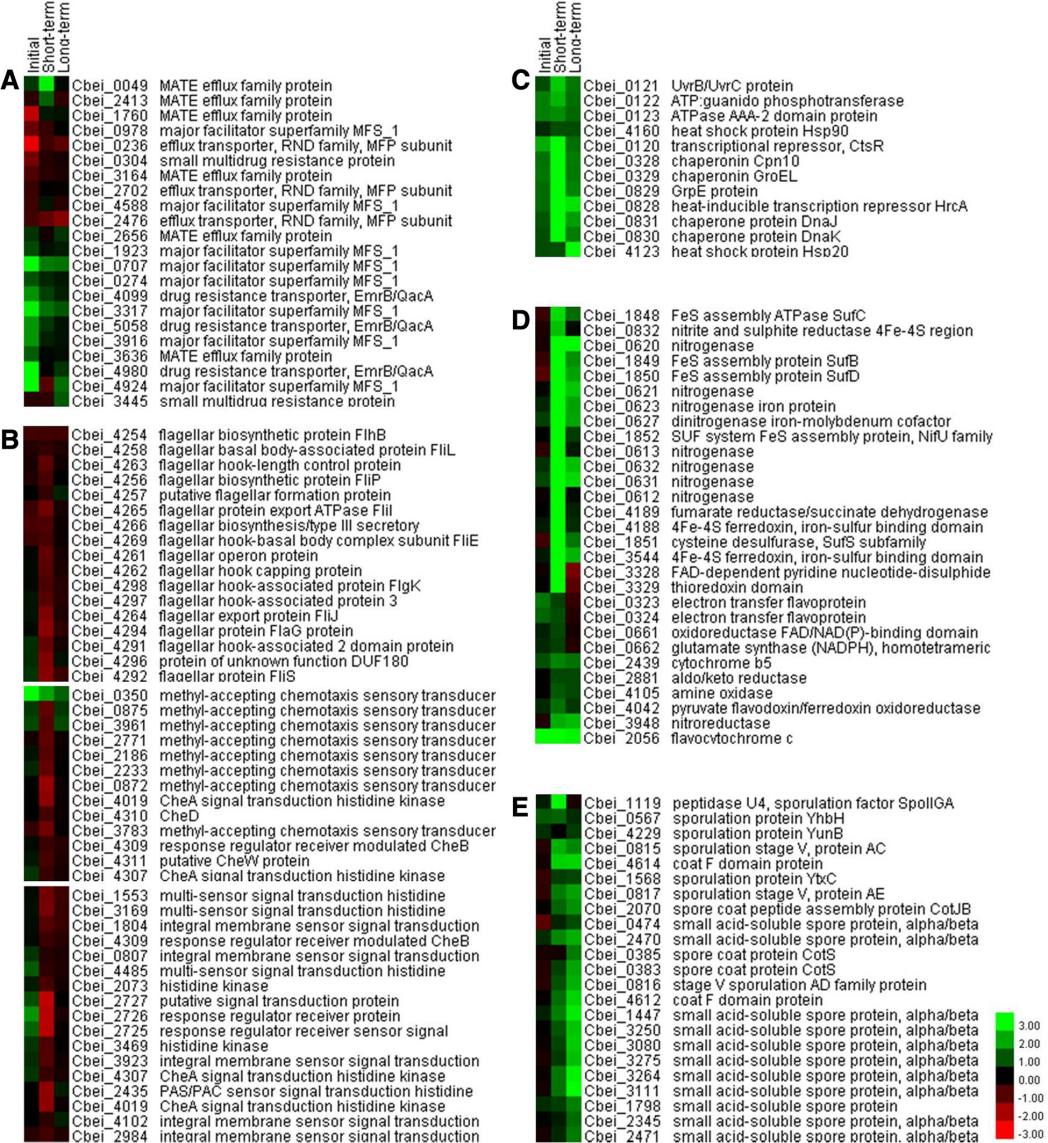
Comparative expression patterns for genes related to **(A)** efflux systems, **(B)** two component systems, including flagellar assembly and chemotaxis, **(C)** heat shock proteins, **(D)** redox reactions, and **(E)** protection of DNA during sporulation. The results shown were those obtained from microarray analysis after *C. beijerinckii* NCIMB 8052 was exposed to 0.5 g/L ferulic acid for varying amounts of time and are presented as the Log_2_ value.

Real-time quantitative PCR (RT-qPCR) was performed to confirm the microarray results obtained were correct using selected genes that were differentially expressed during the challenge with ferulic acid (Figure 2). A group of ten genes representing some of the most highly up- and down-regulated genes within the functionality groups (Figure 3) were selected for validation. As shown in Figure 2 and Additional file 1, the RT-qPCR and microarray analysis results correlated well with each other, with similar gene expression patterns seen for each of the ten individual genes during the different growth stages tested. Within the genes related with the heat shock response, for example, Cbei_0329 and Cbei_0829, which encode the GroEL and GrpE chaperonin proteins, respectively, were evaluated since each exists in different operons. Both were strongly induced when *C. beijerinckii* was exposed to ferulic acid for 4 h (short-term responses), reaching relative expression levels of 8.9 (log_2_ = 3.1) and 21.3 (log_2_ = 4.4), respectively, based on the microarray while similar levels were seen within the RT-qPCR analyses. The other eight genes also displayed similar trends and expression levels in the microarray and RT-qPCR analyses.

### Rapid induction of efflux system-related genes in response to ferulic acid

Within *C. beijerinckii* NCIMB 8052’s ‘instantaneous’ responses to ferulic acid, one down-regulated gene ontology (GO) term, that is, ATPase activity (GO:0016887), was identified (Table 1). More than half of the genes within this term were grouped into a sub-category termed as ‘ATPase activity, coupled’ (GO:0042623), the definition of which is the reactions directly driven by ATP catalysis and include, for example, ion transport across the membrane. Within the culture, ferulic acid is thought to act as an uncoupler due to its hydrophobicity and, therefore, decrease the pH gradient across the membrane of the cell. The abrupt change in the proton motive force brought on by its addition to the culture would affect ATP synthesis [18], and this would in turn lead to a down-regulation of ATP-coupled transport processes. Similar results were seen previously as ion transport-related genes within *E. coli* were also significantly down-regulated by the addition of 0.5 g/L ferulic acid [12]. Moreover, down-regulation of the ATPase activity could be explained by membrane damage and leakage brought on by ferulic acid. It was reported that aromatic acids caused partial membrane disruption and that transmembrane ATPases are affected [19]. Moreover, other transcriptional responses to ferulic acid-induced membrane damage were previously observed in *E. coli* [12] and *L. brevis* [13] cultures. In the *E. coli* study, for example, there was a rapidly mitigated expression of phosphate transport genes when compared to the control presumably since ferulic acid disrupted the membrane and this allowed phosphate, which was present within the media as a buffer, into the cell.

**Table 1:** **GO terms showing significant changes in** ***C. beijerinckii*** **cultures exposed to 0.5 g/L ferulic acid**

**Exposure** ^**a**^	**Category** ^**b**^	**GO ID**	**GO term**	***P*** **value**	**Benjamini** ^**c**^	**Count** ^**d**^
Up-regulated						
I	NS^e^					
S	MF	GO:0048037	Cofactor binding	5.14 E^−05^	0.0102	47
	MF	GO:0016163	Nitrogenase activity	4.47 E^−04^	0.0438	8
L	BP	GO:0006265	DNA topological change	4.12 E^−06^	0.0014	11
Down-regulated						
I	MF	GO:0016887	ATPase activity	3.85 E^−05^	0.0124	38
S	CC	GO:0019861	Flagellum	1.14 E^−08^	4.20 E^−07^	19
	MF	GO:0017076	Purine nucleotide binding	2.41 E^−04^	0.0129	95
	BP	GO:0006935	Chemotaxis	0.0014	0.0316	22
	BP	GO:0006812	Cation transport	4.09 E^−04^	0.0150	20
	BP	GO:0016310	Phosphorylation	0.0011	0.0266	28
	BP	GO:0009309	Amine biosynthetic process	0.0023	0.0444	25
L	NS^e^					

^a^Exposure time to ferulic acid: I, initial; S, short-term; L, long-term; ^b^categories - MF, molecular function; BP, biological process; CC, cellular component; ^c^significant groups were selected based upon Benjamini (<0.05); ^d^the count is the number of genes within the given GO term showing a significant change in expression. The number in parenthesis is the percentage of all genes showing a significant change in their expression levels. ^e^No significant group identified.

An analysis of the Kyoto Encyclopedia of Genes and Genomes (KEGG) pathways also found several genes related to arginine and proline metabolism (cbe00330) up-regulated (Table 2). In fact, this KEGG pathway was the only one present within each of the sampling times as shown in this table. The prevalence of this pathway suggests that *C. beijerinckii* NCIMB 8052 is accumulating proline intracellularly to act as an osmoregulator, as was shown to occur previously in both other bacteria, particularly Gram-positive strains [20], and in yeast [21,22]. Of the genes responding rapidly to ferulic acid, those related with efflux systems showed the greatest changes, with up to 49-fold induction compared to the control culture (Figure 3). Efflux pumps, which are classified into several groups, such as the resistance-nodulation-cell division (RND) superfamily, the small multidrug resistance (SMR) family, the multidrug and toxin extrusion (MATE) protein family, and the major facilitator superfamily (MFS), are reported to increase the tolerance of a microbe to toxic chemicals, including organic solvents and antibiotics [23,24]. In this study, many of the efflux genes showing a fourfold or higher expression level when compared to the control, including the four (Cbei_0707, Cbei_3317, Cbei_4924, and Cbei_4980) showing the greatest inductions (Figure 3, Additional file 2), are members of the MFS. Similar results were seen within tests using *L. brevis* exposed to ferulic acid where uncharacterized major facilitator superfamily permeases were highly expressed [13]. Moreover, two of these genes, Cbei_0707 and Cbei_4924, are linked with MarR family transcriptional regulators, Cbei_0706 and Cbei_4923, respectively [25]. Additional file 2 lists three additional genes as MarR family transcriptional regulators, illustrating the strong and rapid response from various members of this regulon as brought on by an exposure to ferulic acid. The results found here are supported by a previous study with *E. coli* where this regulon was one of the strongest induced by 0.5 g/L ferulic acid [12].

**Table 2:** **KEGG pathways showing significant changes in** ***C. beijerinckii*** **cultures exposed to 0.5 g/L ferulic acid**

**Exposure** ^**a**^	**KEGG ID**	**Pathway definition**	***P*** **value**	**Benjamini** ^**b**^	**Count** ^**c**^
Up-regulated					
I	cbe00330	Arginine and proline metabolism	0.0010	0.0329	11
S	cbe02010	ABC transporters	1.44 E^−07^	9.47 E^−06^	51
	cbe00450	Selenoamino acid metabolism	1.37 E^−05^	4.52 E^−04^	11
	cbe00230	Purine metabolism	1.55 E^−04^	0.0026	23
	cbe00910	Nitrogen metabolism	1.34 E^−04^	0.0030	15
	cbe00920	Sulfur metabolism	2.32 E^−04^	0.0031	9
	cbe00330	Arginine and proline metabolism	0.0032	0.0347	13
L	cbe02010	ABC transporters	1.07 E^−05^	6.09 E^−04^	33
	cbe00330	Arginine and proline metabolism	6.08 E^−04^	0.0172	11
*Down-regulated*					
I	NS^d^				
S	cbe00400	Phenylalanine, tyrosine and tryptophan biosynthesis	1.94 E^−11^	1.01 E^−09^	17
	cbe02040	Flagellar assembly	2.81 E^−10^	7.30 E^−09^	19
L	NS				

^a^Exposure time to ferulic acid: I, initial; S, short-term; L, long-term; ^b^significant groups were selected based upon Benjamini (<0.05); ^c^umber of genes within the given KEGG ID showing a significant change in their expression level; ^d^no significant group identified.

### Modulation of the heat shock and oxidoreductase gene expression levels by ferulic acid

Changes in the *C. beijerinckii* NCIMB 8052 transcriptional levels were more generally pronounced after a 4-h exposure, with many additional genes showing higher expression levels when compared with the 10-min exposure results but slightly fewer showing a repressed expression (Additional files 2, 3, 4, and 5). At this point, the cultures were still in the acidogenic phase, that is, producing acetic acid and butyric acid, and had produced similar amounts of these acids as the unexposed controls. Moreover, the pH of the culture supplemented with ferulic acid (pH = 6.9) was nearly identical to that of the control culture (pH = 7.1). Therefore, the changes in the gene expression levels can be attributed to the presence of ferulic acid and not the physiological conditions of the culture or the shift of the culture from an acidogenic to solventgenic state.

Analyses of the samples taken at this time point found several genes related with flagellar activity were down-regulated within both the GO terms (GO:0019861) and KEGG pathways (cbe02040) (Tables 1 and 2 and Figure 3). Two other GO terms that had several members repressed were chemotaxis (GO:0006935) (Table 1) and the two component sensor activity (GO:0000155), but a Benjamini analysis of the latter was not significant (*P* < 0.05). Chemotaxic regulation of the flagellar activity and, thus, the swimming motion of bacteria is a well-established phenomenon [26]. Furfural, another inhibitor derived from lignocellulosic biomass, was found previously to be toxic to *C. beijerinckii* NCIMB 8052, leading to a repressed expression of two-component signal transduction system and flagellar genes and causing a deficiency within the bacterium’s adaptation machinery [16]. Likewise, it appears that ferulic acid inhibits the same gene clusters, resulting in a reduced ability of *C. beijerinckii* NCIMB 8052 to adapt.

Another group that had a significant change in its expression levels after a 4-h exposure was the genes encoding for the heat shock proteins (HSP). The differential expression of HSPs is a well-documented general stress response when a culture is exposed to various environmental stimuli, such as elevated temperatures or toxic solvents [10,27,28]. This so-called heat shock response aids in host survival under these conditions by helping maintain the cellular proteins within a functional state. Of the several heat shock stimulon classes known, genes present within both class I and III were highly up-regulated in response to ferulic acid stress (Figure 3). Class I heat shock genes, including the *dnaK* and *groE* operons, are molecular chaperones while the class III genes, such as the *clpC* operon, are ATP-dependent proteases. Both of these classes are regulated by repressor proteins, that is, HrcA and CtsR, respectively [29]. In several previous studies with *L. plantarum* and *Bacillus subtilis*, a higher expression of the *clpC* operon was seen after exposure of these strains to *p-*coumarate and salicylate, respectively [30,31]. Similar results were seen when *E. coli* was exposed to 0.25 g/L or greater ferulic acid as the *clpB* gene, one of the genes within the *clp* operon, was strongly induced [12]. Although the presence of a *clpC* operon has not been clearly demonstrated in *C. beijerinckii* NCIMB 8052, the genes within the operon Cbei_0120-Cbei_0123 were found to be orthologous to the genes within the *clpC* operon of *Clostridium acetobutylicum* [32], with Cbei_0120 encoding the transcriptional repressor, CtsR [25]. Expression of these genes was induced by ferulic acid in this study.

The molecular chaperones, however, were more strongly up-regulated than the ATP-dependent proteases. All six genes within the class I HSP grouping (Cbei_0328-Cbei_0329 and Cbei_0828-Cbei_0831) had significantly higher expression levels, fourfold or greater, at 4 h while expression of the *dnaK* operon (Cbei_0828-Cbei_0831) was induced by more than 15-fold when compared with the control cultures (Additional file 4). The other HSPs, however, including Cbei_4123 and Cbei_4160, did not have significantly altered expression levels (<1.5-fold) at this time point (Figure 3). Recently, it was found that unlike *B. subtilis* [29], the *hsp*90 in *C. acetobutylicum* has a HrcA-regulated promoter by which its expression is up-regulated along with other class I genes by several different stresses [33]. In this study, however, expression of the *C. beijerinckii* NCIMB 8052 *hsp*90 gene, that is, Cbei_4160, was not altered by ferulic acid while the other two operons known to be governed by HrcA were significantly induced. Similarly, although the promoter of the *C. acetobutylicum* small HSP has a conserved CtsR operator region [10], the mild increase in expression of Cbei_4123, the small HSP homologue identified in *C. beijerinckii* NCIMB 8052 [34], was not expected as there is a clear and strong induction of the CtsR-regulated *clpC* operon. The reason for the differences in the expression patterns observed between these two strains needs to be studied further.

The transcriptional analysis at this time point found the presence of ferulic acid most significantly affected nitrogenase-related genes, which are classified within the nitrogenase activity (GO:0016163) or oxidoreductase activity (GO:0016732) GO terms (Table 1). Since theseactivities require Fe-S proteins, the genes encoding the Fe-S cluster were also expressed at highly increased level, especially, the operon containing Cbei_1848-Cbei_1852 (Figure 3). It was previously reported that nitrogen fixation genes were induced in *C. acetobutylicum* during an exposure to acetate [35] and since both acetate and ferulic acid act as uncouplers and affect the redox balance, the higher nitrogenase expression levels may result from the bacteria’s attempt to counteract these effects. This idea is supported by the microarray results as several other genes involved in electron transport were also differentially expressed (Figure 3).

Several KEGG pathways were additionally up-regulated by ferulic acid, including ATP-binding cassette (ABC) transporters and sulfur metabolism (Table 2). Induction of ABC transporter-related genes was previously seen in furfural challenged *C. beijerinckii* and ferulic acid stressed *L. brevis* [13,16], as well suggesting that increased expression of the ABC transporters (cbe02010) is a common physiological adaptation of bacteria to stressful environments. In addition, according to a previous study sulfate, assimilation was down-regulated when an *E. coli* culture was exposed to ferulic acid for a short period of time, albeit in a ‘subtle but coordinated’ manner as none of the individual genes was significantly down-regulated [12]. In this study, however, sulfur metabolism (cbe00920) was up-regulated in the short-term exposure samples, suggesting that *C. beijerinckii* might have also experienced mildly reduced sulfur assimilation early on and then expressed both the sulfur metabolism and the ABC transporter genes related with sulfate transport (Cbei_4190-Cbei_4194) later to accommodate for its lack.

### Genes involved in DNA protection during sporulation are up-regulated with long-term ferulic acid exposures

Next, we studied the change in gene expression levels after *C. beijerinckii* NCIMB 8052 was cultured with ferulic acid for a relatively long time. Several studies have previously evaluated the transcriptional responses from fermentative bacterial cultures exposed to inhibitory compounds derived from lignocellulosic biomasses, but the exposure time was only between 10 min and 3 h [13,16,36]. Therefore, an investigation on how a long-term exposure to compounds affects the physiological status and solvent production of the culture is warranted. Both cultures, that is, with and without ferulic acid addition, were analyzed in the late log/early stationary phase when the OD was approximately 5, requiring 9.5 to 12.5 h of growth post ferulic acid addition. We found the cultures were comparable with regard to the pH as well as the solvent and acid yields.

Although many genes had higher expression levels in these samples, many are hypothetical with unknown functions (Additional file 6). Moreover, only four genes were down-regulated fourfold or higher in these cultures (Additional file 7). As such, the responses at this optical density were relatively mild with only one GO term significantly up-regulated and none down-regulated. The GO-term that was induced is related with DNA topological changes (GO:0006265), with six genes showing more than a fourfold induction (Table 1 and Additional file 6). These genes are α/β-type small, acid-soluble spore proteins (SASP) which, along with Ca^2+^-dipicolinic acid, play a key role in DNA protection within spores formed by Gram-positive bacteria [37]. This protein binds and interacts with the cellular DNA to generate a complex that functions in defending the spore and its DNA from physical stresses, such as UV radiation, hydrogen peroxide, or heat [38-40]. As expression of the α/β-type SASPs occurs only in developing spores, late in sporulation [41], a higher expression of the genes encoding these proteins in the ferulic acid-exposed cultures can be interpreted as a faster initiation of sporulation by these cultures than the control cultures. Solventogenic clostridia, such as *C. acetobutylicum* ATCC 824, are known to initiate sporulation with the onset of solventogenesis. The results here, however, suggest that addition of ferulic acid triggers a quicker sporulation than is seen in its absence. This is further evidenced by the cortex and coat formation protein expression patterns, both of which are also expressed in the later stages of sporulation. As shown in Figure 3, the expression levels for both of these genes were also induced. As ferulic acid is clearly toxic to this bacterium, it is not too surprising that the cultures would shift to enter sporulation earlier to survive. Similar responses were seen with other bacterial species exposed to other toxic chemicals [42] as well as in tests with clostridia exposed to syringaldehye or furfural, two inhibitory compounds which like ferulic acid are generated from woody biomass [2,36].

### Enhancement of acetone and butanol production by *C. beijerinckii*/pSAAT-ptb_Gro

**Figure 4:**
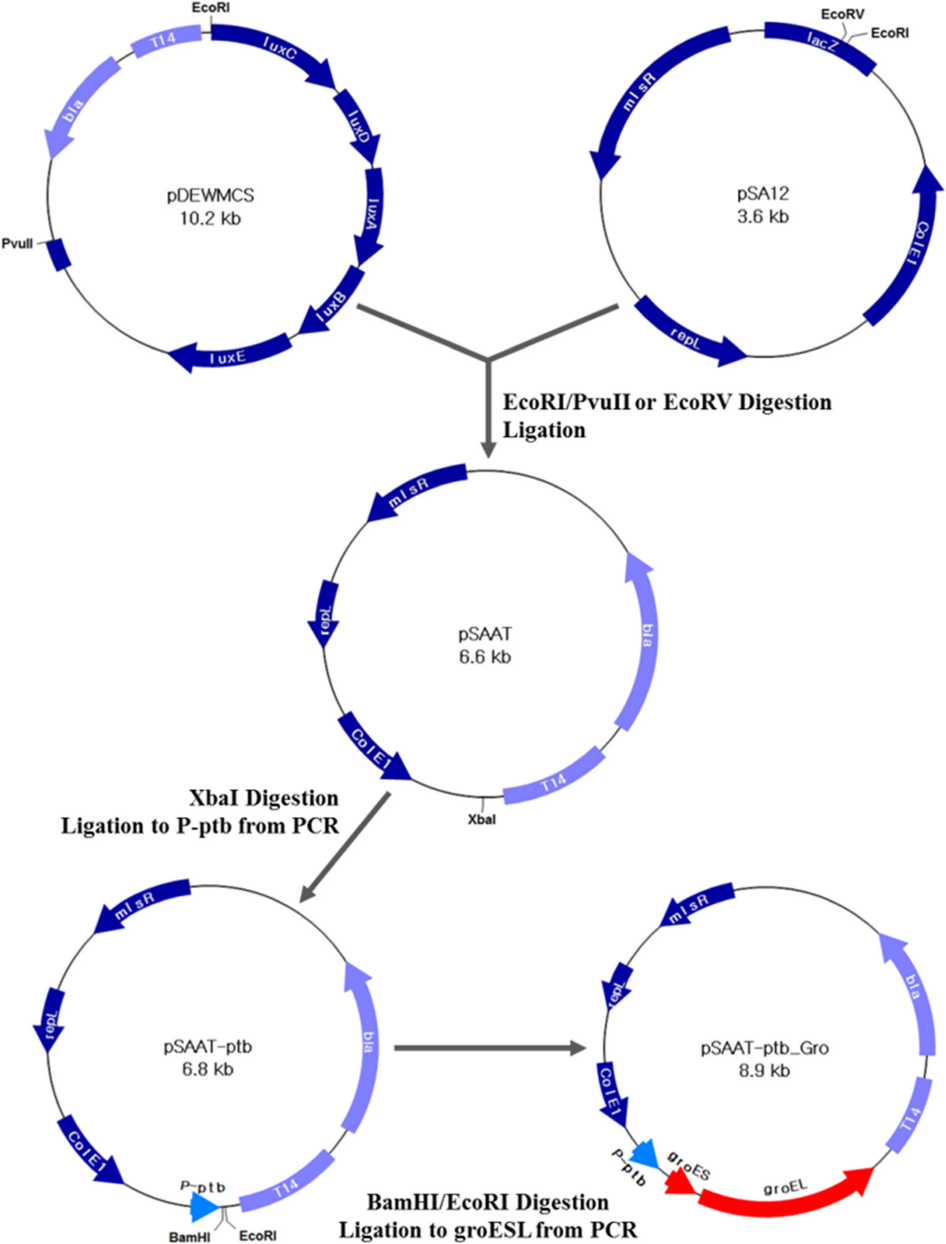
Construction of pSAAT-ptb_Gro. The final shuttle vector was designed so that the expression of *groES*-*groEL* is under the control of the *ptb* promoter and so that it has two origins of replication and antibiotic resistances for use within both *E. coli* and *Clostridium*. The plasmids are drawn with directions of the relevant genes and the restriction sites used in each step of construction.

**Figure 5:**
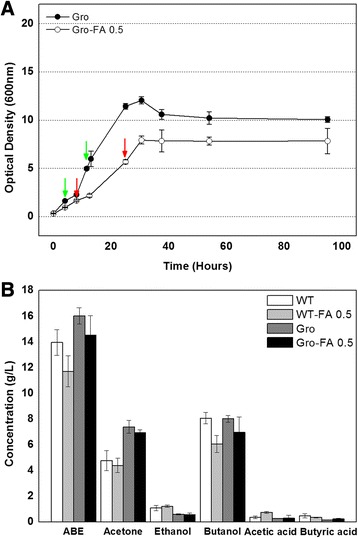
Effects of ferulic acid on the **(A)** growth of and **(B)** solvent production by the recombinant strain of *C. beijerinckii.* For the RT-qPCR analysis in Figure 6, the cells were sampled at two points as indicated by the arrows. And the final products from fermentation by the recombinant strain were compared with those from wild type strain.

Based upon the above analyses, the *groE* operon genes were selected for overexpression in *C. beijerinckii*. Overexpression of HSPs has been used to relieve several stresses, such as to heat or toxic solvents [27,43,44], while several groups recently reported that they also improve tolerance to one or multiple alcohols [45,46]. Using the same principle, we attempted to clone the *groE* operon genes from *C. beijerinckii* but were unsuccessful as this operon seems to be toxic to *E. coli*. However, we were able to clone the *groESL* genes from *C. acetobutylicum* ATCC 824 and, consequently, performed the tolerance tests with this homologous gene cluster. For this, plasmid pSAAT-ptb_Gro (Figure 4) was introduced into *C. beijerinckii*. To understand the effects of overexpressing *groESL* on the metabolic activity of *C. beijerinckii*, the growth and solvent production by both the wild-type and recombinant strain harboring pSAAT-ptb_Gro were monitored in the absence or presence of ferulic acid. As shown in Figure 5A, and similar with the wild-type strain in Figure 1, the growth of the recombinant strain, labeled as ‘Gro’, was inhibited by ferulic acid. Moreover, the growth rate and the maximum optical density were lower than those of wild-type strain (Figure 1), even in the absence of ferulic acid, a result that was also observed with *C. acetobutylicum* previously [33]. This lower biomass can be attributed to the larger number and activity of GroES and GroEL proteins within the recombinant strain since they require ATP when aiding in protein refolding [47].

**Figure 6:**
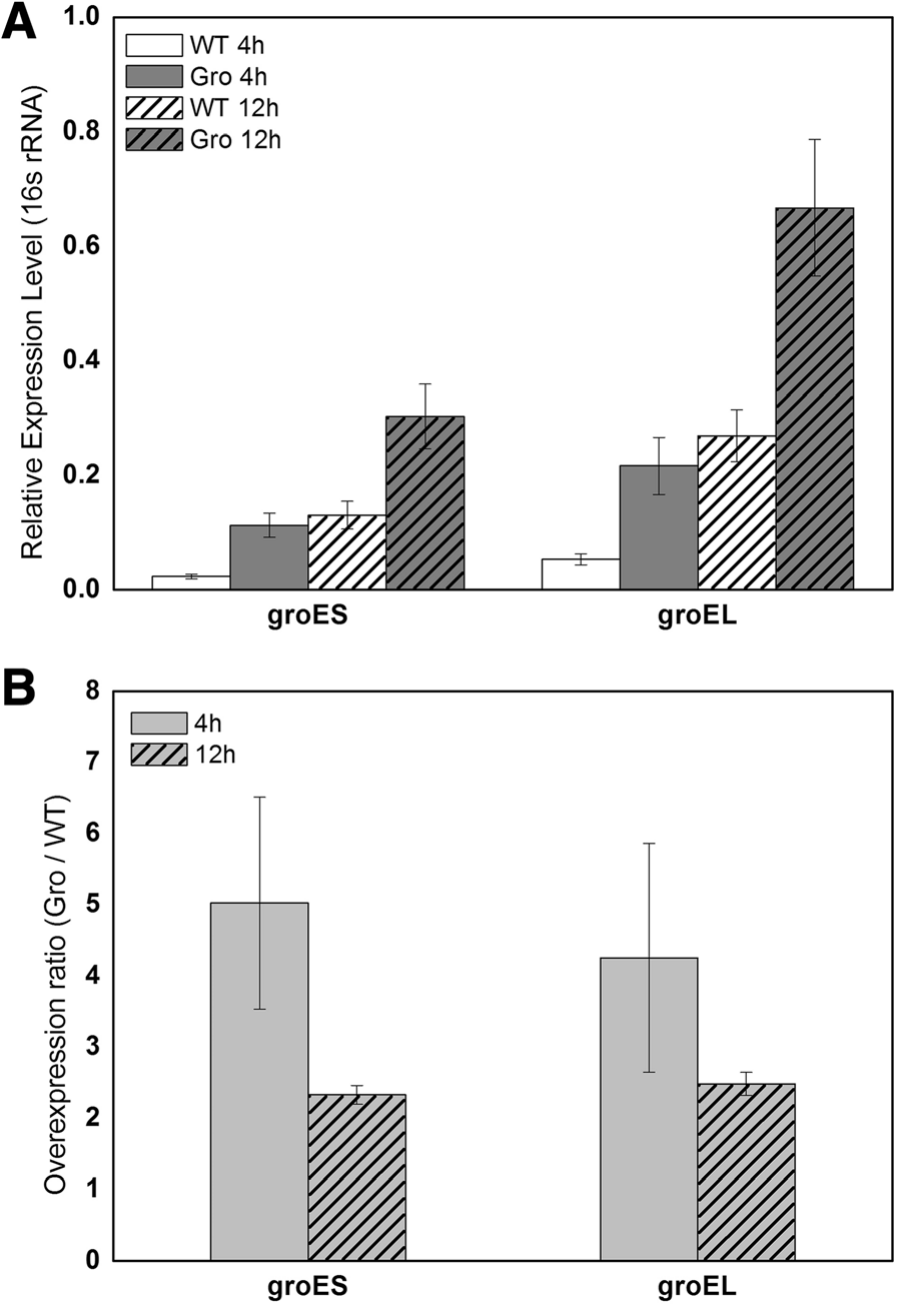
*groES* and *groEL* expression levels within the wild-type and recombinant (*C. beijerinckii* strains. The results are presented as **(A)** the relative expression level (normalized using the 16 s rRNA concentrations) for each point and **(B)** the fold increase within the recombinant strain as calculated based on the values in **(A)**.

To confirm that *groESL* expression within this strain was higher, RT-qPCR analysis was performed using RNA prepared from two sampling points as indicated by arrows in Figure 5A. As shown in Figure 6, a 2.5- to 5-fold higher relative expression was seen for both *groES* and *groEL* within *C. beijerinckii*/pSAAT-ptb_Gro. Although the expression levels of the genes were generally higher for the second sampling point, a greater ratio was obtained from the earlier samples and this can be partially explained by the stronger activation of *ptb* promoter during the acidogenic stage of *C. beijerinckii* growth.

The solvents produced by the recombinant strain were next analyzed and compared with those from the wild-type strain under matching fermentative conditions (Figure 5B). Without addition of ferulic acid, the wild-type culture and that of *C. beijerinckii*/pSAAT-ptb_Gro produced identical amounts of butanol, while the recombinant strain produced approximately 3 g/L more acetone. One possible explanation for the modified solvent and acid profiles from the recombinant strain is that the higher production of GroES and GroEL helps to stabilize the thiolase protein. This protein is responsible for conversion of acetyl-CoA to acetoacetyl-CoA and, if more stable, would draw carbon away from acetate and ethanol thereby lowering their yields. Moreover, when producing acetone, the acetate and butyrate that was produced could be utilized through the activity of coenzyme-A transferase (CoAT) leading to acetyl-CoA and butyrl-CoA, respectively. By regenerating acetate and butyrate through acetyl-phosphate and butyrl-phosphate, this bacterium can then produce additional ATP.

In one previous study, the overexpression of *groESL* under the *thl* promoter inside *C. acetobutylicum* likewise improved the amount of acetone produced but the butanol yield also increased [33]. Although it is not certain why the tests performed here gave different results, it may be partially due to the promoter used for *groESL* expression since the *thl* promoter is constitutively activated whereas the *ptb* promoter is primarily activated during the acidogenic phase. Nevertheless, the butanol yield in the recombinant culture was 15% higher than the wild-type culture when both were challenged with ferulic acid (Figure 5B), with yields of 6.98 and 6.07 g/L, respectively. However, the solvent production using the recombinant strain was still not as high as the wild-type without ferulic acid, suggesting that the tolerance of strain may be enhanced further.

## Conclusions

This study investigated the transcriptional responses from *C. beijerinckii* NCIMB 8052 under ferulic acid stress. From microarray analysis, several gene groups were identified to show significant change for each status. As the instant response, efflux system-related genes were highly up-regulated, including the gene encoding major facilitator superfamily and its MarR family regulator, which agrees well with previous study with *E. coli* exposed to ferulic acid. When *C. beijerinckii* NCIMB 8052 was exposed to ferulic acid for more time until OD reached 1.4, heat shock protein coding genes were induced, where molecular chaperones were shown to be higher than ATP-dependent proteases, while the genes involved in two component signal transduction system were down-regulated. Nitrogenase was also expressed and acted to make up redox balance which was destroyed by ferulic acid interruption. Addition of ferulic acid also induced the expression of genes related with DNA protection during sporulation within *C. beijerinckii*. Based upon the analysis, *groES* and *groEL* genes, encoding heat shock proteins, were selected for overexpression within *C. beijerinckii* to construct a strain tolerant to lignin-hydrolysate-related compounds. This constructed recombinant strain produced more acetone and butanol even under ferulic acid stressed condition, finally providing the potential as an applicant strain for biomass hydrolysate fermentation.

## Methods

### Bacterial strains and growth condition

All the strains and plasmids used in this study are listed in Table 3. *C. beijerinckii* NCIMB 8052 spores were stored at 4°C until needed. The spores were germinated by heat shocking them at 75°C for 10 min. After chilling on ice, 1 mL was inoculated into 20 mL of reinforced clostridia medium (RCM) (Difco, Detroit, MI, USA) and incubated anaerobically within a serum bottle at 37°C for 12 h. The cells were sub-cultured (5%) into fresh media and grown 9 h to reach an optical density (OD) at 600 nm of 2.0. For the chemical exposure tests, 5 ml of this culture was transferred into 50 mL of P2 media with or without 0.5 g/L of ferulic acid and cultured for 84 h. P2 media was prepared as previously described [48]. For growth of *C. beijerinckii* harboring the recombinant plasmid, erythromycin was used at a concentration of 20 μg/L to retain the plasmid during growth. For the plasmid construction, *E. coli* DH5α was used as a host strain and was grown in Luria broth (LB) (Difco, Detroit, MI, USA) or on LB agar plates. Ampicillin 100 μg/L was added to medium when needed.

**Table 3:** **Bacterial strains and plasmids used in this study**

**Strain or plasmid**	**Relevant characteristics**	**Reference**
Strains		
*C. beijerinckii* NCIMB 8052	Wild type	
*C. acetobutylicum* ATCC 824	Wild type	
*E. coli* DH5α	*fhuA2* Δ *(argF-lacZ) U169 phoA glnV44* Φ*80* Δ *(lacZ) M15 gyrA96 recA1 relA1 endA1 thi-1 hsdR17*	
Plasmids		
pDEWMCS	Modified pDEW201 plasmid, MCS contains restriction sites for *Not*I, *Xho*I, *BamH*I, *Xma*I, *Xba*I, *EcoR*V, and *Kpn*I; Amp^R^	
pSA12	Em^r^, shuttle vector, ColE1 *ori*, pIM13 *oriII*	15
pSAAT	Em^R^, Amp^R^, shuttle vector, ColE1 *ori*, pIM13 *oriII,* T1-4	This study
pSAAT-ptb	Em^R^, Amp^R^, shuttle vector, ColE1 *ori*, pIM13 *oriII,* T1-4, P*ptb,*	This study
pSAAT-ptb_Gro	Em^R^, Amp^R^, shuttle vector, ColE1 *ori*, pIM13 *oriII*, T1-4, P*ptb*-*groES*-*groEL*	This study

Amp^R^, amipicillin resistant; Em^R^, erythromycin resistant.

### Product analysis

Cell growth was analyzed by measuring the optical density at 600 nm using a BioPhotometer plus (Eppendorf, Hamburg, Germany). The solvent and acid concentrations generated by the culture were measured using a gas chromatograph (Agilent Technologies 7890A, Santa Clara, CA, USA) equipped with a flame ionization detector. Nitrogen gas was used as the carrier gas, and the column was a BP21 capillary column (30 m × 0.25 mm × 0.25 μm) (SGE Analytical Science, Melrose Park, NSW, Australia). The temperatures of the injector and detector were kept at 250°C, and the oven temperature was programmed to be held initially at 50°C for 1 min, followed by an increase from 50°C to 240°C at a rate of 20°C/min. The glucose concentration was determined using high-performance liquid chromatography (Agilent Technologies 1200 series, Santa Clara, CA, USA) with a Bio-Rad Aminex HPX-87P column (300 × 7.8 mm) (Hercules, CA, USA). The temperature for the column was 80°C and HPLC-grade water (DAEJUNG Chemicals, Siheung-si, Gyeonggi-do, Korea) was used as the mobile phase at a flow rate of 0.6 mL/min.

### RNA isolation

For RNA isolation, 10 mL of the culture was harvested by centrifugation at 4,000 rpm for 10 min at 4°C. The cell pellet was resuspended and treated with 20 mg/mL lysozyme at 37°C for 4 min in SET buffer (25% sucrose, 50 mM EDTA pH 8.0, 50 mM Tris-HCl pH 8.0) [49]. To each sample, 1 mL of Trizol (Ambion, Foster City, CA, USA) and 200 μL of chloroform-isoamyl alcohol (Sigma, St. Louis, MO, USA) were added and the samples were incubated at room temperature. After centrifugation at 12,000 *g* for 15 min at 4°C, the upper aqueous phase was transferred to new tubes containing an equal volume of 70% ethanol. To purify the RNA from the sample, this mixture was transferred to a spin column (RNeasy Mini Kit, Qiagen, Valencia, CA, USA), and further purification steps were performed according to the manufacturer’s protocol. The RNA samples were then treated with DNase I (Thermo Scientific, Waltham, MA, USA) to completely remove any genomic DNA remaining. The RNA quality was assessed using a Bioanalyzer 2100 (Agilent Technologies, Santa Clara, CA, USA) while the quantity and concentration of RNA was determined with a NanoDrop 2000 (Thermo Scientific, Waltham, MA, USA).

### Microarray experiment and analysis

The microarray chips for this study were constructed by Agilent Technologies. A total of 5,009 protein coding *C. beijerinckii* NCIMB 8052 genes were examined. The arrays were designed to have three identical replicates probe for each gene. The samples taken at OD 0.3 and 1.4 were analyzed in triplicate, but those for OD 5 were tested in duplicate, requiring 16 arrays in total with the respective unexposed controls. All microarray experiments were performed according to the protocol provided for the Agilent One-Color Microarray-Based Exon Analysis. Fluorescent cRNA for each sample was generated using 100 ng of RNA using a Low Input Amp WT Labeling Kit, One color (Agilent Technologies, Santa Clara, CA, USA) and then purified using the RNeasy Mini Kit (Qiagen). When the labeling reaction was prepared, the RNA Spike-In Kit, One color (Agilent Technologies, Santa Clara, CA, USA) was also used as an internal control. After quantification, 600 ng of the cRNA was used for hybridization with the Gene Expression Hybridization Kit (Agilent Technologies, Santa Clara, CA, USA). Hybridization was conducted at 65°C for 17 h, followed by washing and drying of the chip using the Gene Expression Wash Buffer Kit (Agilent Technologies, Santa Clara, CA, USA). Finally, the microarray slides were scanned with an Agilent G2565CA Microarray Scanner System (Agilent Technologies, Santa Clara, CA, USA).

The features, which were scanned at a 5 μm pixel size, were extracted and analyzed with the Agilent Feature Extraction software v10.5 (Agilent Technologies, Santa Clara, CA, USA). The data obtained was then normalized using the 75th percentile value of non-control signals on each array as provided by the program. Any signals below the negative control were excluded while the other signals were averaged using the replicates present within the arrays. Afterwards, the relative expression levels were calculated by dividing the adjusted values for the test samples, that is, those exposed to ferulic acid, by those for control samples at each OD point. Finally, genes showing a 1.5-fold or greater change (with *P* < 0.05) were selected to conduct functional enrichment analysis according to their gene ontology terms and KEGG pathways using the DAVID bioinformatics tool [50]. Treeview v.1.6 was used to visualize the gene expression trends according to the OD [51]. The microarray data and results are available online at the Gene Expression Omnibus (http://www.ncbi.nlm.nih.gov/geo) under the accession number GSE67244.

### Real-time quantitative PCR analysis

To validate these results, several genes showing strong up- or down-regulated expression levels based upon the microarray results were selected for confirmation by real-time quantitative PCR analysis. Total RNA samples were prepared as described above, and 1 μg was used to synthesize the cDNA using the RevertAid First Strand cDNA Synthesis Kit (Thermo Scientific, Waltham, MA, USA) according to the manufacturer’s suggested protocol.

For the RT-qPCR reaction, 1 μL of the cDNA samples was used and the volumes of Power SYBR® Green PCR Master Mix (Applied Biosystems, Warrington, UK) and the primers were scaled according to the manufacturer’s recommendation. Primers were designed to have an annealing temperature (Tm) of 58°C ± 1°C and a 5′ additional sequence flap for the better responses (Additional file 8) [52]. The reaction was programmed on a LightCycler® 480 Real-Time PCR System (Roche, Indianapolis, IN, USA) as described previously [12], except that the initial denaturation at 95°C was extended to 10 min. For the analysis, the gene concentrations within each reaction were normalized based upon the 16 s rRNA concentration; 16 s rRNA gene concentration within each reaction was determined using *C. beijerinckii* NCIMB 8052 genomic DNA standards, and this value was used to normalized the concentration of each test gene.

### Construction of a new shuttle vector and overexpression plasmid, pSAAT-ptb_Gro

All enzymes used in this study, including the restriction enzymes, Phusion High-fidelity PCR polymerase and T4 ligase, were purchased from Thermo Scientific (Waltham, MA, USA). A new *E. coli*-*Clostridium* shuttle vector was constructed by inserting the approximately 3 kb fragment from pDEWMCS [53] digested with *Pvu*II and *Eco*RI into pSA12 [18] digested with *EcoRV* and *EcoRI*, giving pSAAT. Afterwards, the promoter region from the *C. beijerinckii* NCIMB 8052 *ptb* gene was amplified with the primers listed in Additional file 2, thereby introducing an *XbaI* site. The amplicon was cloned into pSAAT after both were digested with *XbaI* and then transformed into competent *E. coli* DH5α (RBC Biosciences, New Taipei City, Taiwan). After incubation at 37°C overnight on LB agar plates containing 100 μg/L ampicillin, the correct colonies were confirmed by PCR using the primers, Ptb-p RX and SeqR, which also determined if the clones had the proper orientation. The final shuttle vector was designated pSAAT-ptb.

Amplification of the *groE* gene was carried out by PCR using genomic DNA from *C. acetobutylicum* ATCC 824 with the primers shown in Additional file 2. The PCR fragment and pSAAT-ptb were both digested with *Eco*RI and *Bam*HI and ligated together. The ligation mixture was transformed into competent *E. coli* DH5α (RBC Biosciences) and spread on LB agar plates containing ampicillin. This plate was incubated at 25°C for several days before colonies were fully grown. This temperature was chosen since no colonies were obtained when the plates were incubated at 37°C, a result that was seen previously [33]. The plasmid, pSAAT-ptb_Gro, was isolated from a correct clone.

### Transformation into *C. beijerinckii* NCIMB 8052

Plasmid pSAAT-ptb_Gro was transformed into *C. beijerinckii* NCIMB 8052 using electroporation using the modified method described previously [54]. Briefly, *C. beijerinckii* cultures grown anaerobically overnight in RCM were transfered into tryptone-glucose-yeast extract (TGY) medium [25] and grown to an OD of 0.6. The cells at this state were harvested by centrifugation at 3,000 *g* for 10 min at 4°C and washed with 1 volume of ice-cold electroporation buffer (ETB) (270 mM sucrose, 5 mM NaH_2_PO_4_, pH 7.4). After centrifugation as above, the cell pellet was resuspended in ice-cold ETB to a final volume that was 1/25th that of the initial cell volume. This suspension was immediately used for electroporation; all of the procedures of which were performed under anaerobic conditions.

A total of 2 μg plasmid DNA was mixed with 400 μL of the competent cell suspension as prepared above, and this was then incubated for 10 min on ice. The mixture was added to an electrotransformation cuvette (4 mm gap width), and electroporation was performed using an Electroporator 2510 (Eppendorf, Hamburg, Germany) with the voltage set to 2 kV. The cells were immediately transferred to 8 mL of TGY medium and incubated for 4 h at 37°C. The cells were then centrifuged and resuspended with a small volume of TGY medium and plated on a TGY agar plate containing 20 μg/L erythromycin.

## Additional files

Additional file 1: Figure S1. Comparison between expression level of ten representative genes as determined by microarray and RT-qPCR analyses. The data is presented according to the three growth states. Additional file 2: Table S1. Genes up-regulated fourfold or higher when *C. beijerinckii* NCIMB 8052 cultures exposed to ferulic acid at 0.5 g/L reached an OD of 0.3. Additional file 3: Table S2. Genes down-regulated fourfold or greater when *C. beijerinckii* NCIMB 8052 cultures exposed to ferulic acid at 0.5 g/L reached an OD of 0.3. Additional file 4: Table S3. Genes up-regulated fourfold or higher when *C. beijerinckii* NCIMB 8052 cultures exposed to ferulic acid at 0.5 g/L reached an OD of 1.4. Additional file 5: Table S4. Genes down-regulated fourfold or greater when *C. beijerinckii* NCIMB 8052 cultures exposed to ferulic acid at 0.5 g/L reached an OD of 1.4. Additional file 6: Table S5. Genes up-regulated fourfold or higher when *C. beijerinckii* NCIMB 8052 cultures exposed to ferulic acid at 0.5 g/L reached an OD of 5. Additional file 7: Table S6. Genes down-regulated fourfold or higher when *C. beijerinckii* NCIMB 8052 cultures exposed to ferulic acid at 0.5 g/L reached an OD of 5. Additional file 8: Table S7. Primers used in this study.

## Abbreviations

ABC: ATP-binding cassette
ATCC: American Type Culture Collection
CoAT: coenzyme-A transferase
GO: gene ontology
HSP: heat shock protein
KEGG: Kyoto Encyclopedia of Genes and Genomes
LB: Luria broth
MATE: multidrug and toxin extrusion
MFS: major facilitator superfamily
NCIMB: National Collection of Industrial, Marine and Food Bacteria
OD600: optical density at 600 nm
RCM: reinforced clostridia medium
RND: resistance-nodulation-cell division
RT-qPCR: real-time quantitative PCR
SASP: small, acid-soluble spore proteins
TGY: tryptone-glucose-yeast extract medium
