# Supplementary material for: Analysis of Clostridium beijerinckii NCIMB 8052’s transcriptional response to ferulic acid and its application to enhance the strain tolerance
Source: Biotechnol Biofuels. 2015 Apr 16;8:68. doi: 10.1186/s13068-015-0252-9 (PMC4406174; doi:10.1186/s13068-015-0252-9)

Supplementary Figure 1. Comparison between expression level of ten representative genes as determined by microarray and RT-qPCR analyses. The data is presented according to the three growth states.

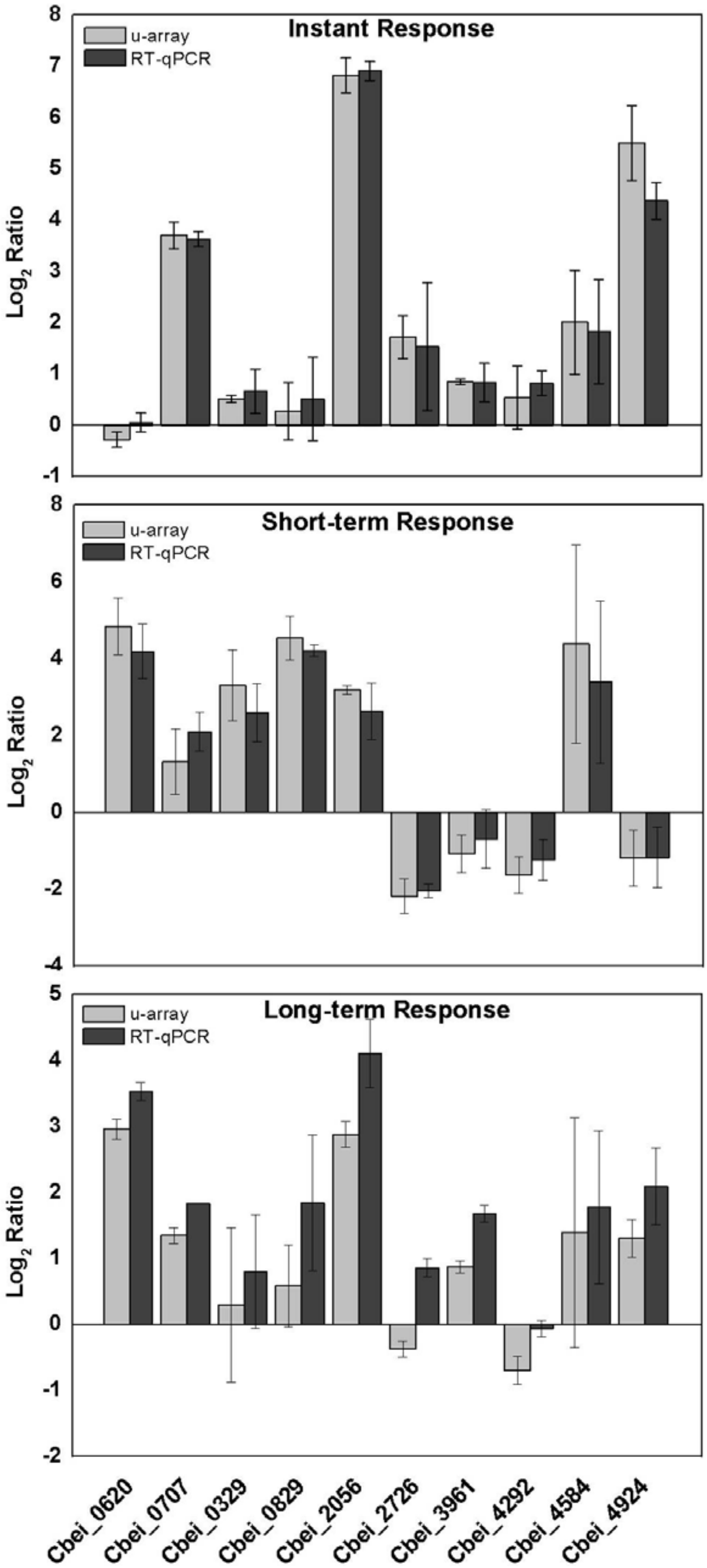

Supplement: Additional file 1: Figure S1. — Comparison between expression level of ten representative genes as determined by microarray and RT-qPCR analyses. The data is presented according to the three growth states. [file 13068_2015_252_MOESM1_ESM.pdf]
