# Supplementary material for: Analysis of Clostridium beijerinckii NCIMB 8052’s transcriptional response to ferulic acid and its application to enhance the strain tolerance
Source: Biotechnol Biofuels. 2015 Apr 16;8:68. doi: 10.1186/s13068-015-0252-9 (PMC4406174; doi:10.1186/s13068-015-0252-9)
Supplement: Additional file 2: Table S1. — Genes up-regulated fourfold or higher when C. beijerinckii NCIMB 8052 cultures exposed to ferulic acid at 0.5 g/L reached an OD of 0.3. [file 13068_2015_252_MOESM2_ESM.pdf]

Supplementary Table 1. Genes up-regulated 4-fold or higher when *C. beijerinckii* NCIMB 8052 cultures exposed to ferulic acid at 0.5 g/L reached an OD of 0.3.

**Gene symbol**	**Gene name**	**Fold-change**	**P-value**
Cbei_0348	transcriptional regulator, MarR family	9.23	0.014
Cbei_0349	nitrogen-fixing NifU domain protein	55.38	0.013
Cbei_0350	methyl-accepting chemotaxis sensory transducer	60.34	0.023
Cbei_0387	spore coat protein CotS	4.16	0.019
Cbei_0596	transcriptional regulator, DeoR family	6.30	0.026
Cbei_0707	major facilitator superfamily MFS_1	13.16	0.019
Cbei_0792	quinolinate synthetase complex, A subunit	4.85	0.001
Cbei_1842	transcriptional regulator, DeoR family	5.52	0.047
Cbei_1843	1-phosphofructokinase	9.25	0.039
Cbei_1844	PTS system, fructose subfamily, IIC subunit	8.73	0.046
Cbei_1927	NADH:flavin oxidoreductase/NADH oxidase	10.47	0.013
Cbei_2055	NADPH-dependent FMN reductase	84.01	0.005
Cbei_2056	flavocytochrome c	115.15	0.021
Cbei_2057	protein of unknown function DUF1304	4.62	0.011
Cbei_2158	rubredoxin-type Fe(Cys)4 protein	8.81	0.021
Cbei_2522	ribonucleoside-triphosphate reductase,	4.64	0.042
Cbei_2870	substrate-binding region of ABC-type glycine	15.35	0.003
Cbei_2871	glycine betaine/L-proline ABC transporter,	14.73	0.012
Cbei_3130	regulatory protein GntR, HTH	5.77	0.010
Cbei_3298	putative galactoside ABC transporter	6.59	0.026
Cbei_3299	ABC transporter related	9.25	0.003
Cbei_3300	ABC transporter related	9.03	0.009
Cbei_3301	transcriptional regulator, MarR family	5.44	0.000
Cbei_3317	major facilitator superfamily MFS_1	7.11	0.007
Cbei_3339	conserved hypothetical protein	4.71	0.042
Cbei_3340	hypothetical protein	4.19	0.009
Cbei_3477	conserved hypothetical protein	4.05	0.079
Cbei_3892	conserved uncharacterized protein	5.68	0.009
Cbei_3893	transcriptional regulator, MarR family	6.07	0.008
Cbei_4084	mannonate dehydratase	5.31	0.056
Cbei_4101	secretion protein HlyD family protein	6.30	0.021
Cbei_4403	hypothetical protein	4.32	0.045
Cbei_4521	ornithine carbamoyltransferase	4.86	0.006
Cbei_4584	ABC transporter related	4.78	0.034
Cbei_4923	transcriptional regulator, MarR family	16.07	0.005
Cbei_4924	major facilitator superfamily MFS_1	49.26	0.023
Cbei_4980	drug resistance transporter, EmrB/QacA	22.30	0.001
