# Supplementary material for: Analysis of Clostridium beijerinckii NCIMB 8052’s transcriptional response to ferulic acid and its application to enhance the strain tolerance
Source: Biotechnol Biofuels. 2015 Apr 16;8:68. doi: 10.1186/s13068-015-0252-9 (PMC4406174; doi:10.1186/s13068-015-0252-9)
Supplement: Additional file 3: Table S2. — Genes down-regulated fourfold or greater when C. beijerinckii NCIMB 8052 cultures exposed to ferulic acid at 0.5 g/L reached an OD of 0.3. [file 13068_2015_252_MOESM3_ESM.pdf]

Supplementary Table 2. Genes down-regulated 4-fold or greater when *C. beijerinckii* NCIMB 8052 cultures exposed to ferulic acid at 0.5 g/L reached an OD of 0.3.

Gene symbol	Gene name	Fold-change	P-value
Cbei_0236	efflux transporter, RND family, MFP subunit	-6.63	0.013
Cbei_0237	ABC transporter related	-6.47	0.007
Cbei_0238	protein of unknown function DUF214	-9.00	0.008
Cbei_0239	cystathionine gamma-synthase	-7.99	0.020
Cbei_0240	cystathionine gamma-synthase	-4.71	0.001
Cbei_0590	integral membrane sensor signal transduction	-4.71	0.033
Cbei_0591	glycoside hydrolase, family 25	-4.27	0.073
Cbei_0760	membrane spanning protein	-4.83	0.005
Cbei_1445	protein of unknown function DUF1002	-5.50	0.040
Cbei_1517	uncharacterized membrane protein, putative	-4.50	0.049
Cbei_1760	MATE efflux family protein	-4.73	0.034
Cbei_1888	GRound-like protein	-8.45	0.001
Cbei_2115	DNA mismatch repair protein MutS domain protein	-4.93	0.007
Cbei_2558	hypothetical protein	-4.30	0.016
Cbei_2947	UvrD/REP helicase	-4.75	0.008
Cbei_3063	protein of unknown function DUF156	-5.70	0.000
Cbei_3064	heavy metal translocating P-type ATPase	-4.71	0.012
Cbei_3354	NADPH-dependent FMN reductase	-4.39	0.003
Cbei_3760	ABC transporter related	-8.09	0.016
Cbei_3761	transport system permease protein	-4.09	0.048
Cbei_3801	formate dehydrogenase, alpha subunit	-6.94	0.005
Cbei_3906	nitroreductase	-4.31	0.041
Cbei_3910	transcriptional regulator, RpiR family	-4.31	0.046
Cbei_3911	hypothetical protein	-4.72	0.034
Cbei_3912	VanZ family protein	-6.43	0.047
Cbei_4194	sulfate ABC transporter, periplasmic	-7.13	0.025
Cbei_4202	abortive infection protein	-4.88	0.008
Cbei_4367	DNA ligase-like protein	-4.66	0.005
Cbei_4790	hypothetical protein	-5.98	0.075
Cbei_4902	lipoprotein signal peptidase	-6.12	0.011
