# Supplementary material for: Analysis of Clostridium beijerinckii NCIMB 8052’s transcriptional response to ferulic acid and its application to enhance the strain tolerance
Source: Biotechnol Biofuels. 2015 Apr 16;8:68. doi: 10.1186/s13068-015-0252-9 (PMC4406174; doi:10.1186/s13068-015-0252-9)
Supplement: Additional file 4: Table S3. — Genes up-regulated fourfold or higher when C. beijerinckii NCIMB 8052 cultures exposed to ferulic acid at 0.5 g/L reached an OD of 1.4. [file 13068_2015_252_MOESM4_ESM.pdf]

Supplementary Table 3. Genes up-regulated 4-fold or higher when *C. beijerinckii* NCIMB 8052 cultures exposed to ferulic acid at 0.5 g/L reached an OD of 1.4.

Gene symbol	Gene name	Fold-change	P-value
Cbei_0017	histidine kinase	4.20	0.003
Cbei_0049	MATE efflux family protein	7.19	0.018
Cbei_0055	methionyl-tRNA synthetase	8.84	0.065
Cbei_0056	hydrolase, TatD family	4.24	0.085
Cbei_0120	transcriptional repressor, CtsR	4.29	0.008
Cbei_0239	cystathionine gamma-synthase	15.40	0.005
Cbei_0240	cystathionine gamma-synthase	12.35	0.017
Cbei_0256	4'-phosphopantetheinyl transferase	4.77	0.073
Cbei_0328	chaperonin Cpn10	6.81	0.006
Cbei_0329	chaperonin GroEL	11.28	0.010
Cbei_0330	Type II secretory pathway pseudopilin PulG-like	6.06	0.100
Cbei_0331	inosine-5'-monophosphate dehydrogenase	4.17	0.046
Cbei_0343	two component regulator three Y domain protein	4.26	0.050
Cbei_0349	nitrogen-fixing NifU domain protein	5.62	0.001
Cbei_0596	transcriptional regulator, DeoR family	5.29	0.089
Cbei_0604	NMT1/THI5-like protein	41.90	0.042
Cbei_0605	binding-protein-dependent transport systems	55.96	0.027
Cbei_0606	ABC transporter related	90.10	0.009
Cbei_0607	heavy metal translocating P-type ATPase	39.76	0.046
Cbei_0608	heavy metal transport/detoxification protein	7.63	0.068
Cbei_0609	O-acetylhomoserine/O-acetylserine sulfhydrylase	92.22	0.017
Cbei_0610	ABC transporter related	44.79	0.034
Cbei_0611	ABC-type nitrate/sulfonate/bicarbonate transport	141.84	0.008
Cbei_0612	nitrogenase	31.39	0.047
Cbei_0613	nitrogenase	107.70	0.042
Cbei_0614	binding-protein-dependent transport systems	22.95	0.030
Cbei_0615	binding-protein-dependent transport systems	68.94	0.036
Cbei_0617	binding-protein-dependent transport systems	29.22	0.060
Cbei_0618	binding-protein-dependent transport systems	22.95	0.015
Cbei_0620	nitrogenase	30.81	0.011
Cbei_0621	nitrogenase	52.64	0.011
Cbei_0622	cysteine synthase	52.30	0.001
Cbei_0623	nitrogenase iron protein	59.82	0.011
Cbei_0624	ABC transporter related	53.13	0.018
Cbei_0625	binding-protein-dependent transport systems	61.59	0.015
Cbei_0626	NLPA lipoprotein	28.97	0.006
Cbei_0627	dinitrogenase iron-molybdenum cofactor	65.19	0.026
Cbei_0628	radical SAM domain protein	60.90	0.009
Cbei_0629	cystathionine gamma-synthase	42.44	0.029
Cbei_0630	pyridoxal-5'-phosphate-dependent protein, beta	42.57	0.046
Cbei_0631	nitrogenase	171.88	0.016

Supplementary Table 3. (continued)

Gene symbol	Gene name	Fold-change	P-value
Cbei_0632	nitrogenase	204.97	0.037
Cbei_0657	methyltransferase type 11	5.47	0.084
Cbei_0766	O-acetylhomoserine/O-acetylserine sulfhydrylase	42.86	0.018
Cbei_0812	anti-sigma-factor antagonist	5.24	0.077
Cbei_0813	putative anti-sigma regulatory factor,	6.43	0.065
Cbei_0814	RNA polymerase, sigma 28 subunit, FliA/WhiG	6.56	0.059
Cbei_0815	sporulation stage V, protein AC	4.20	0.024
Cbei_0828	heat-inducible transcription repressor HrcA	23.33	0.037
Cbei_0829	GrpE protein	24.25	0.041
Cbei_0830	chaperone protein DnaK	15.20	0.011
Cbei_0831	chaperone protein DnaJ	15.70	0.001
Cbei_1119	peptidase U4, sporulation factor SpoIIGA	7.54	0.094
Cbei_1431	seryl-tRNA synthetase	8.56	0.061
Cbei_1474	branched-chain amino acid transport system II	5.67	0.054
Cbei_1764	inner-membrane translocator	10.76	0.089
Cbei_1765	ABC transporter related	9.74	0.092
Cbei_1767	extracellular ligand-binding receptor	7.84	0.063
Cbei_1848	FeS assembly ATPase SufC	6.15	0.024
Cbei_1849	FeS assembly protein SufB	14.81	0.033
Cbei_1850	FeS assembly protein SufD	16.78	0.026
Cbei_1851	cysteine desulfurase, SufS subfamily	19.89	0.067
Cbei_1852	SUF system FeS assembly protein, NifU family	23.47	0.030
Cbei_1977	diaminopropionate ammonia-lyase	4.00	0.086
Cbei_1987	regulatory protein, LysR	4.19	0.026
Cbei_2055	NADPH-dependent FMN reductase	12.65	0.008
Cbei_2056	flavocytochrome c	9.13	0.001
Cbei_2126	aspartyl-tRNA synthetase	5.83	0.055
Cbei_2155	hypothetical protein	5.95	0.080
Cbei_2594	conserved hypothetical protein	4.15	0.040
Cbei_2595	hypothetical protein	4.34	0.008
Cbei_2608	aspartate 1-decarboxylase	8.27	0.049
Cbei_2609	pantoate--beta-alanine ligase	9.86	0.030
Cbei_2610	3-methyl-2-oxobutanoate	12.17	0.006
Cbei_2611	conserved hypothetical protein	10.65	0.016
Cbei_2654	3-oxoacid CoA-transferase, A subunit	4.45	0.023
Cbei_3543	O-acetylhomoserine	32.14	0.024
Cbei_3544	4Fe-4S ferredoxin, iron-sulfur binding domain	28.79	0.072
Cbei_3709	cobalt/cobalamin transport protein CbiN	4.17	0.030
Cbei_3710	cobalamin biosynthesis protein CbiM	4.06	0.040
Cbei_3712	cobalt ABC transporter, inner membrane subunit	5.18	0.002
Cbei_3948	nitroreductase	4.15	0.041
Cbei_3981	extracellular solute-binding protein, family 5	5.34	0.020
Cbei_4329	hypothetical protein	6.45	0.088

Supplementary Table 3. (continued)

Gene symbol	Gene name	Fold-change	P-value
Cbei_4524	NADPH-dependent FMN reductase	4.01	0.025
Cbei_4578	accessory gene regulator B	4.40	0.053
Cbei_4583	hypothetical protein	20.24	0.079
Cbei_4584	ABC transporter related	39.98	0.042
Cbei_4587	hypothetical protein	20.92	0.070
Cbei_4769	hypothetical protein	4.87	0.065
Cbei_4798	PfkB domain protein	68.53	0.004
Cbei_4799	sugar isomerase (SIS)	36.57	0.014
Cbei_4800	extracellular solute-binding protein, family 3	22.01	0.001
Cbei_4926	uncharacterised conserved protein UCP033563	6.13	0.064
Cbei_4927	D-isomer specific 2-hydroxyacid dehydrogenase,	5.82	0.048
Cbei_5014	hypothetical protein	4.28	0.000
Cbei_5015	StbA family protein	16.09	0.001
Cbei_5043	inner-membrane translocator	4.09	0.020
Cbei_5044	inner-membrane translocator	4.13	0.063
