# Supplementary material for: Analysis of Clostridium beijerinckii NCIMB 8052’s transcriptional response to ferulic acid and its application to enhance the strain tolerance
Source: Biotechnol Biofuels. 2015 Apr 16;8:68. doi: 10.1186/s13068-015-0252-9 (PMC4406174; doi:10.1186/s13068-015-0252-9)
Supplement: Additional file 5: Table S4. — Genes down-regulated fourfold or greater when C. beijerinckii NCIMB 8052 cultures exposed to ferulic acid at 0.5 g/L reached an OD of 1.4. [file 13068_2015_252_MOESM5_ESM.pdf]

Supplementary Table 4. Genes down-regulated 4-fold or greater when *C. beijerinckii* NCIMB 8052 cultures exposed to ferulic acid at 0.5 g/L reached an OD of 1.4.

Gene symbol	Gene name	Fold-change	P-value
Cbei_1455	putative cell wall binding repeat-containing	-6.34	0.061
Cbei_1456	peptidase S8 and S53, subtilisin, kexin,	-4.44	0.060
Cbei_1826	RNA polymerase, sigma 28 subunit, FliA/WhiG	-4.92	0.007
Cbei_1827	hypothetical protein	-4.02	0.033
Cbei_1828	homocysteine S-methyltransferase	-6.02	0.007
Cbei_1829	phosphoglycerate mutase	-5.49	0.054
Cbei_2411	conserved hypothetical protein	-5.90	0.081
Cbei_2725	response regulator receiver sensor signal	-4.44	0.049
Cbei_2726	response regulator receiver protein	-4.40	0.034
Cbei_2727	putative signal transduction protein	-4.79	0.040
Cbei_3286	hypothetical protein	-10.55	0.094
Cbei_3472	oxidoreductase FAD/NAD(P)-binding domain	-4.08	0.073
Cbei_4197	FeoA family protein	-4.80	0.027
Cbei_4696	putative cell wall binding repeat-containing	-4.77	0.071
Cbei_4698	cell wall hydrolase/autolysin	-8.40	0.069
Cbei_4718	putative cell wall binding repeat-containing	-17.60	0.082
Cbei_4719	putative cell wall binding repeat-containing	-4.42	0.052
