# Supplementary material for: Analysis of Clostridium beijerinckii NCIMB 8052’s transcriptional response to ferulic acid and its application to enhance the strain tolerance
Source: Biotechnol Biofuels. 2015 Apr 16;8:68. doi: 10.1186/s13068-015-0252-9 (PMC4406174; doi:10.1186/s13068-015-0252-9)
Supplement: Additional file 6: Table S5. — Genes up-regulated fourfold or higher when C. beijerinckii NCIMB 8052 cultures exposed to ferulic acid at 0.5 g/L reached an OD of 5. [file 13068_2015_252_MOESM6_ESM.pdf]

Supplementary Table 5. Genes up-regulated 4-fold or higher when *C. beijerinckii* NCIMB 8052 cultures exposed to ferulic acid at 0.5 g/L reached an OD of 5.

Gene symbol	Gene name	Fold-change	P-value
Cbei_0349	nitrogen-fixing NifU domain protein	7.64	0.010
Cbei_0620	nitrogenase	7.78	0.018
Cbei_0621	nitrogenase	5.12	**0.122**
Cbei_0622	cysteine synthase	13.70	**0.232**
Cbei_0625	binding-protein-dependent transport systems	5.67	0.072
Cbei_0628	radical SAM domain protein	4.77	0.087
Cbei_0631	nitrogenase	5.98	0.003
Cbei_0632	nitrogenase	4.76	0.133
Cbei_0641	hypothetical protein	4.74	0.175
Cbei_0816	stage V sporulation AD family protein	4.28	0.246
Cbei_0869	short-chain dehydrogenase/reductase SDR	5.10	0.274
Cbei_0889	hypothetical protein	4.02	0.022
Cbei_0991	hypothetical protein	6.94	0.196
Cbei_0993	delta-lactam-biosynthetic de-N-acetylase	4.15	0.116
Cbei_1127	phosphate binding protein	4.80	0.036
Cbei_1333	conserved hypothetical protein	9.87	0.035
Cbei_1447	small acid-soluble spore protein, alpha/beta	6.95	0.246
Cbei_1472	nitroreductase family protein	6.46	0.185
Cbei_1617	conserved hypothetical protein	11.47	0.121
Cbei_1619	conserved hypothetical protein	9.57	0.059
Cbei_1620	hypothetical protein	9.06	0.026
Cbei_1621	RecT protein	5.04	0.091
Cbei_1622	beta-lactamase domain protein	13.30	0.015
Cbei_1623	primosome, DnaD subunit	7.67	0.013
Cbei_1624	hypothetical protein	10.96	0.015
Cbei_1625	hypothetical protein	7.04	0.040
Cbei_1626	protein of unknown function DUF1064	9.39	0.050
Cbei_1627	conserved hypothetical protein	5.73	0.314
Cbei_1628	hypothetical protein	6.00	0.110
Cbei_1632	DNA methylase N-4/N-6 domain protein	4.82	0.007
Cbei_1638	hypothetical protein	10.45	0.026
Cbei_1639	hypothetical protein	4.89	0.074
Cbei_1641	hypothetical protein	12.60	0.014
Cbei_1642	hypothetical protein	21.90	0.031
Cbei_1644	hypothetical protein	15.45	0.081
Cbei_1645	hypothetical protein	17.47	0.048
Cbei_1647	hypothetical protein	13.08	0.058
Cbei_1648	hypothetical protein	6.71	0.049
Cbei_1649	phage XkdN-like protein	19.09	0.103
Cbei_1650	phage tail tape measure protein, TP901 family	12.22	0.048
Cbei_1651	hypothetical protein	6.54	0.123
Cbei_1653	hypothetical protein	19.83	0.028

Supplementary Table 5. (continued)

Gene symbol	Gene name	Fold-change	P-value
Cbei_1656	baseplate J family protein	8.13	0.037
Cbei_1657	hypothetical protein	11.13	0.084
Cbei_1659	hypothetical protein	5.19	0.142
Cbei_1663	Ig domain protein, group 2 domain protein	4.60	0.093
Cbei_1664	conserved hypothetical protein	5.76	0.081
Cbei_1916	protein of unknown function DUF541	4.90	0.071
Cbei_2055	NADPH-dependent FMN reductase	15.57	0.024
Cbei_2056	flavocytochrome c	7.39	0.013
Cbei_2470	small acid-soluble spore protein, alpha/beta	4.25	0.105
Cbei_2522	ribonucleoside-triphosphate reductase,	5.54	0.052
Cbei_2598	amine oxidase	4.30	0.192
Cbei_2599	cysteine desulfurase family protein	4.14	0.132
Cbei_3080	small acid-soluble spore protein, alpha/beta	5.32	0.304
Cbei_3111	small acid-soluble spore protein, alpha/beta	8.76	0.259
Cbei_3250	small acid-soluble spore protein, alpha/beta	5.07	0.105
Cbei_3264	small acid-soluble spore protein, alpha/beta	7.35	0.245
Cbei_3385	conserved hypothetical protein	4.27	0.099
Cbei_3386	putative periplasmic ligand-binding sensor	15.49	0.147
Cbei_3388	putative tail fiber-related protein	9.10	0.148
Cbei_3389	hypothetical protein	5.77	0.179
Cbei_3390	baseplate J family protein	6.18	0.139
Cbei_3391	hypothetical protein	6.51	0.102
Cbei_3392	hypothetical protein	9.68	0.149
Cbei_3393	phage-like element pbsx protein XkdQ	16.86	0.155
Cbei_3394	hypothetical protein	10.90	0.020
Cbei_3395	Phage-related protein	15.05	0.074
Cbei_3396	Phage XkdN-like protein	11.72	0.043
Cbei_3397	phage-like element pbsx protein XkdM	16.93	0.101
Cbei_3398	phage-like element pbsx protein XkdK	11.42	0.044
Cbei_3399	hypothetical protein	10.22	0.005
Cbei_3401	hypothetical protein	7.13	0.002
Cbei_3544	4Fe-4S ferredoxin, iron-sulfur binding domain	4.35	0.068
Cbei_3732	AAA ATPase	4.55	0.093
Cbei_3733	hypothetical protein	4.49	0.083
Cbei_3734	hypothetical protein	4.77	0.005
Cbei_3735	transcriptional regulator, XRE family	7.23	0.124
Cbei_3739	metallophosphoesterase	4.81	0.081
Cbei_3948	nitroreductase	4.66	0.165
Cbei_4611	hypothetical protein	8.66	0.265
Cbei_4612	coat F domain protein	5.67	0.282
Cbei_4613	hypothetical protein	13.17	0.115
Cbei_4614	coat F domain protein	5.76	0.077
Cbei_4767	hypothetical protein	17.57	0.236
Cbei_4768	hypothetical protein	25.65	0.267

Supplementary Table 5. (continued)

Gene symbol	Gene name	Fold-change	P-value
Cbei_4769	hypothetical protein	16.65	0.268
Cbei_4770	hypothetical protein	18.05	0.229
Cbei_4834	catalase	6.92	0.199
