# Supplementary material for: Analysis of Clostridium beijerinckii NCIMB 8052’s transcriptional response to ferulic acid and its application to enhance the strain tolerance
Source: Biotechnol Biofuels. 2015 Apr 16;8:68. doi: 10.1186/s13068-015-0252-9 (PMC4406174; doi:10.1186/s13068-015-0252-9)
Supplement: Additional file 7: Table S6. — Genes down-regulated fourfold or higher when C. beijerinckii NCIMB 8052 cultures exposed to ferulic acid at 0.5 g/L reached an OD of 5. [file 13068_2015_252_MOESM7_ESM.pdf]

Supplementary Table 6. Genes down-regulated 4-fold or higher when *C. beijerinckii* NCIMB 8052 cultures exposed to ferulic acid at 0.5 g/L reached an OD of 5.

Gene symbol	Gene name	Fold-change	P-value
Cbei_0232	binding-protein-dependent transport systems	-4.30	0.143
Cbei_3336	drug resistance transporter, EmrB/QacA	-7.47	0.319
Cbei_3972	amino acid permease-associated region	-4.20	0.103
Cbei_4367	DNA ligase-like protein	-4.40	0.172
