# Supplementary material for: Analysis of Clostridium beijerinckii NCIMB 8052’s transcriptional response to ferulic acid and its application to enhance the strain tolerance
Source: Biotechnol Biofuels. 2015 Apr 16;8:68. doi: 10.1186/s13068-015-0252-9 (PMC4406174; doi:10.1186/s13068-015-0252-9)
Supplement: Additional file 8: Table S7. — Primers used in this study. [file 13068_2015_252_MOESM8_ESM.pdf]

Supplementary Table 7. Primers used in this study

Primer	Sequence
**RT-qPCR analysis**	
16s rRNA F	AATAAATCATAA CGCACAAGCAGCGGAGCAT
16s rRNA R	AATAAATCATAA AACCCAACATCTCACGACACGA
Cbei_0328 F	AATAAATCATAA ACCACTTGGTGAAAGAGTAG
Cbei_0328 R	AATAAATCATAA CCTTTACTTCTGTTCCAGCA
Cbei_0329 F	AATAAATCATAA TTTGGGGAAGATGCAAGAAG
Cbei_0329 R	AATAAATCATAA GCACCCATGTTTTCATATGG
Cbei_0328 F2	AATAAATCATAATGAACATTAAACCACTTGGTGA
Cbei_0328 R2	AATAAATCATAATCCAGGTCCTACTGCAAC
Cbei_0329 F2	AATAAATCATAAAAGAAGATATAGCTAGAGTTGC
Cbei_0329 R2	AATAAATCATAACCTCTATCGAATTGCATACC
Cbei_0620 F	AATAAATCATAAATGTAACGCAGGTCATGCTT
Cbei_0620 R	AATAAATCATAAATCTTCCTATTGTAGCCGGA
Cbei_0707 F	AATAAATCATAAGATCGTTTTGGAGAAGGAATGGG
Cbei_0707 R	AATAAATCATAAACCCCCAAGCACGGCTAGT
Cbei_0829 F	AATAAATCATAATAGAAGCTGCTGAGGAAGAT
Cbei_0829 R	AATAAATCATAATACATCTACATATGCATCGCT
Cbei_2056 F	AATAAATCATAA ACAGAAGGACTTGCAGATGT
Cbei_2056 R	AATAAATCATAAGGTTAACTCCTGCAAGCTTA
Cbei_2726 F	AATAAATCATAAAGTCGCTTTTTGCAGGTAGT
Cbei_2726 R	AATAAATCATAACCATTCTAACTGTGTTAGGAG
Cbei_3961 F	AATAAATCATAACTAGCTCATCCTACTAAGAC
Cbei_3961 R	AATAAATCATAA CTGAACATAGCTCACAAGCA
Cbei_4292 F	AATAAATCATAATATGTTAGTTGAAGGTGCGG
Cbei_4292 R	AATAAATCATAACCATCCTGCTTGTGTTGTAT
Cbei_4584 F	AATAAATCATAATGGATGAGCCTTTTACTGGA
Cbei_4584 R	AATAAATCATAAGAGTTTTGCCCTTATCAATCA
Cbei_4924 F	AATAAATCATAATTATACGGGCAGGAGCTAAA
Cbei_4924 R	AATAAATCATAAAGAGTTTGCATAGGAGAGAG
**Plasmid construction**	
Ptb-p FX	TGCTCTAGAATAGAAGATATATTATATTACGTTCGTGTTGTGA
Ptb-p RX	GCATCTAGAAATCAATGCTATGAATATTTCTTTATACCTT
A_groE FB	GTAGGATCCGCCAAAATTAAGTTTATACTAAAAG
A_groE RE	GTAGAATTCAATGCACTCTTATTACATTAATC
Seq F	GAAAGGGGGATGTGCTGCAAGGCG
Seq R	GCTTCCGGCTCGTATGTTGTGTGG
